# Bibliometric and visual analysis in the field of tea in cancer from 2013 to 2023

**DOI:** 10.3389/fonc.2023.1296511

**Published:** 2024-01-11

**Authors:** Yuanchu Lian, Xiating Li, Ying Lan, Zonghuai Li, Xiaoxin Lin, Jing’an Huang, Bo Zhang, Zhongwen Feng

**Affiliations:** ^1^ Scientific Research Center, Guilin Medical University, Guilin, China; ^2^ Lingui Clinical College, Guilin Medical University, Guilin, China; ^3^ Department of Pharmacy, Guangxi Academy of Medical Sciences and the People’s Hospital of Guangxi Zhuang Autonomous Region, Nanning, China

**Keywords:** bibliometric, tea, cancer, polyphenols, molecular mechanisms

## Abstract

**Objective:**

Tea has been utilized in cancer research and is progressively gaining wider recognition, with its roles in cancer prevention and treatment being increasingly affirmed. The objective of this study is to investigate the current state and research hotspots in the field of tea’s involvement in cancer research from 2013 to 2023, aiming to offer reference and direction for future studies.

**Methods:**

We analyzed 4,789 articles published between 2013 and 2022 from the Web of Science database using VOSviewer, R software, and CiteSpace software.

**Result:**

Tea-related cancer research showed an overall upward trend, with China leading in publications, followed by the United States, India, Japan, and Italy. China also had significant international collaborations, notably with Harvard University and the Egyptian Knowledge Bank. The ‘Journal of Agricultural and Food Chemistry’ was the most cited journal. Key topics included ‘green tea,’ ‘cancer,’ ‘*in vitro*,’ ‘oxidative stress,’ and ‘apoptosis.’ Research focused on tea’s pharmacological effects, anticancer properties, mechanisms of natural compounds (e.g., polyphenols and EGCG), antioxidant and antimicrobial properties, and molecular mechanisms in cancer treatment.

**Conclusion:**

Tea’s potential as an anti-cancer medication is gaining global recognition. Our study provides a comprehensive analysis of tea-related cancer research from 2013 to 2023, guiding future investigations in this field.

## 1 Introduction

Cancer is the leading cause of death and a major obstacle to increasing global life expectancy (1). In 2020 alone, 19.3 million new cancer cases are expected to occur, leading to nearly 10 million cancer deaths worldwide. Cancer, by any measure, continues to exert a substantial toll on both morbidity and mortality worldwide (2). The primary goal of cancer treatment is to extend the lives of patients and improve their quality of life (3). Various approaches are employed in cancer treatment, including surgery (4), chemotherapy (5), immunotherapy (6), radiotherapy (7), and a recent innovative method that harnesses cytokine networks to combat cancer. This novel approach has emerged as a result of a deeper understanding of the tumor microenvironment and more effective immunotherapeutic strategies (8). Regrettably, chemotherapy resistance persists as a formidable challenge in the realm of cancer research and treatment (9). Furthermore, the recurrence of malignant tumors represents a pivotal obstacle to achieving a cure for cancer, profoundly affecting the long-term survival and quality of life of afflicted individuals (4). Consequently, the global community continues to grapple with the daunting task of cancer treatment and prevention.

Tea, with its origins rooted in ancient China, boasts a history spanning thousands of years. Over this extensive period, tea has established itself as a globally cherished beverage, enjoyed by people of all ages on a daily basis (10). Tea exhibits a diverse range of types, stemming from different levels of fermentation applied to tea leaves, with green tea, oolong tea, and black tea representing some of the principal categories (11). Regardless of the type, all teas originate from the tender leaves, stems, and buds of the Camellia sinensis plant. Tea comprises a rich array of compounds, including polyphenolic compounds like epicatechin and catechin, flavonol glycosides, L-theanine, theaflavin, theobromine, and various volatile organic substances (12–14). Notably, tea has important medicinal properties and its plant extracts have preventive and therapeutic effects on a wide range of diseases, including cardiovascular diseases, malignant tumors, digestive disorders, and metabolic disorders such as obesity and diabetes (15).

Tea abounds in polyphenolic flavonoids, including catechins (16). Many flavonoids exhibit various beneficial properties, including antioxidant effects, the ability to scavenge free radicals, prevention of coronary heart disease, hepatoprotective properties, anti-inflammatory actions, and even anticancer potential. The potential influence of tea on the risk of developing various types of malignant tumors has undergone extensive research and has shown its effectiveness in the treatment of various cancers (17, 18). Numerous studies have emphasized that the green tea polyphenol (-)-epigallocatechin-3-gallate (EGCG) can attenuate radiation-induced intestinal damage and restore the intestinal ecological balance that has been disrupted by radiation, thereby reducing radiation toxicity during radiotherapy (19). Furthermore, certain flavonoids have shown promise in combating viral infections (20). A multitude of research findings support the notion that green tea catechins (GTCs) may exert potent anticancer and chemopreventive effects on various types of cancers, including hepatocellular carcinoma. Additionally, several experiments have demonstrated that GTCs can improve abnormal metabolic function and prevent the development of precancerous lesions (21). Therefore, tea exhibits significant potential and advantages in the treatment and prevention of cancer. However, it is important to acknowledge that the use of EGCG in cancer treatment still faces challenges such as low bioavailability, limited solubility, and an uncertain therapeutic window (22). Consequently, the field of tea’s application in cancer treatment still has a long road ahead (23).

Pritchard (1969) originally defined bibliometrics as “the application of mathematical and statistical methods to books and other forms of communication,” and later by Hawkins (2001) as “the quantitative analysis of bibliographic attributes within a body of literature” (24). Through bibliometrics, researchers can analyze research hotspots and development trends within published literature, facilitating a rapid understanding of research frontiers and hot topics within a specific field (25, 26). While a substantial amount of literature exists on the topic of tea in the context of cancer, there has been a noticeable absence of bibliometric analyses within the oncology domain. Consequently, the research trends and hotspots concerning tea in the cancer field remain unclear. To address this gap, we utilized R software, VOSviewer, and CiteSpace to perform a comprehensive analysis of relevant literature in the intersection of tea and cancer. Our aim was to elucidate the current research status of this field, identify research hotspots and emerging trends, and provide valuable references for future studies.

## 2 Materials and methods

### 2.1 Data collection

On July 23, 2023, the data utilized for this study were acquired and downloaded from WoSCC (Guilin Medical University’s subscription version). We used the following search formula: (TS= (cancer* OR tumor* OR neoplasm OR neoplasia* OR “malignant neoplasm*” OR malignancy OR malignancies OR “Benign Neoplasm*”)) AND TS= (tea) AND DOP=(2013-07-23/2023-07-23)) AND DT=(Article OR Review)) AND LA=(English). After eliminating irrelevant literature, we observed 4,789 papers (with no duplicates). The collected papers were stored in a plain text format and exported as complete records, including their corresponding cited references.

### 2.2 Data analysis

To analyze annual publications, we utilized Origin 2018. Additionally, we employed several tools for data visualization and scientific knowledge mapping, including the bibliometrix package in R software (version 4.3.1, http://www.bibliometrix.org), VOSviewer (version 1.6.18), and CiteSpace (version 6.1.4).

We used VOSviewer to create visual representations of the co-author network of countries/institutions, co-citation analysis of sources, and co-occurrence of keywords. For the co-authorship network analysis, we applied the following parameters: The number of documents must be ≥5 for a country and ≥15 for an institution. In the co-citation analysis of sources, we set the number of citations a source must have ≥ 400. In addition, in the keyword co-occurrence analysis, the minimum number of occurrences of a keyword was ≥12, and keywords such as “cancer”, “tea” and “tumor” were excluded. To retrieve journal impact factors (IFs), we referenced the Journal Citation Reports (JCR) for the year 2023.

## 3 Results

### 3.1 General landscapes of included documents on tea in cancer

**Figure 1:**
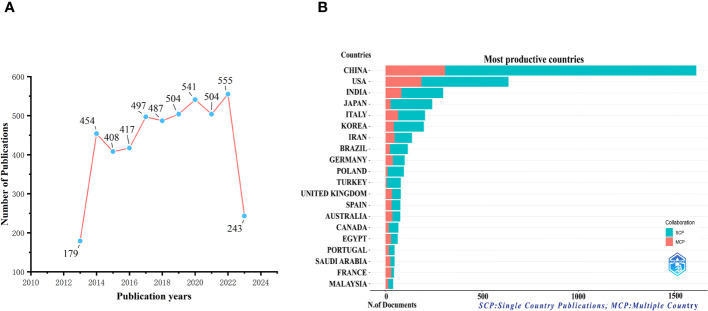
Trends in annual publications in the field of tea in cancer, 2013-2023. **(A)** Trends in publishing outputs, by year. **(B)** Distribution of corresponding authors’ countries and cooperation.

We collected a total of 4,789 documents from WoSCC with no duplicates. **Figure 1A** depicts the trend in publications related to tea’s research in cancer, showing both increases and decreases. The most significant growth occurred between 2013 and 2014. Subsequently, from 2014 to 2022, there was a slight rise in the volume of relevant literature, peaking at 555 publications in 2022. As of July 23, 2023, a total of 243 new documents had been published.

**Figure 2:**
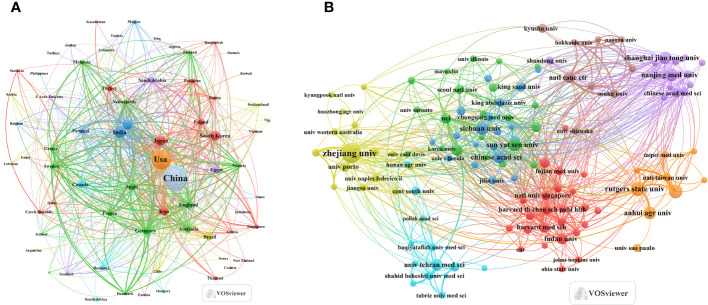
Map of Tea in Cancer Countries/Regions and Institutions, 2013-2023. **(A)** Map of cooperation between different countries. **(B)** Map of cooperation between different institutions.

According to the countries of corresponding authors, we observed that China (n = 1,605) had been the most prolific, followed by the USA (n = 637), India (n = 299), Japan (n = 242), and Italy (n = 204). Within the group of top five nations by article publication volume, Japan had only 10.7% of multinational publications, significantly lower than the other four countries. Notably, Italy had the highest number of multinational publications, accounting for 31.9% (see **Figure 1B**, **Table 1**). Moreover, **Figure 2A** illustrates that China has engaged in the most extensive international cooperation in the field of tea research in cancer. Additionally, **Figure 2B** and **Table 2** demonstrate that Harvard University (n = 183) and the Egyptian Knowledge Bank (n = 158) serve as notable collaboration centers.

**Table 1:** The corresponding author of tea in cancer in the relevant countries.

Country	Articles	SCP	MCP	Freq	MCP_Ratio
China	1605	1298	307	0.335	0.191
Usa	637	451	186	0.133	0.292
India	299	218	81	0.062	0.271
Japan	242	216	26	0.051	0.107
Italy	204	139	65	0.043	0.319
Korea	198	155	43	0.041	0.217
Iran	137	91	46	0.029	0.336
Brazil	115	92	23	0.024	0.2
Germany	98	60	38	0.02	0.388
Poland	95	85	10	0.02	0.105
Turkey	79	73	6	0.016	0.076
United Kingdom	79	46	33	0.016	0.418
Spain	77	46	31	0.016	0.403
Australia	76	41	35	0.016	0.461
Canada	66	50	16	0.014	0.242
Egypt	63	35	28	0.013	0.444
Portugal	46	29	17	0.01	0.37
Saudi Arabia	45	21	24	0.009	0.533
France	44	15	29	0.009	0.659
Malaysia	39	25	14	0.008	0.359

**Table 2:** Most relevant affiliations of tea in the field of cancer research.

Affiliation	Articles
Harvard University	184
Egyptian Knowledge Bank (Ekb)	158
Udice-French Research Universities	130
University Of Alabama Birmingham	123
University Of California System	115
University Of Alabama System	99
Chinese University Of Hong Kong	89
Harvard T.H. Chan School Of Public Health	86
Zhejiang University	78
Chinese Academy Of Sciences	77
Zhejiang University	76
University Of Texas System	71
Tehran University Of Medical Sciences	68
Rutgers State University New Brunswick	66

### 3.2 Journals and co-cited journals

**Figure 3:**
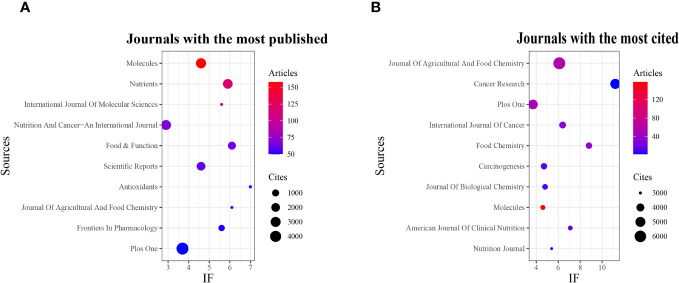
Journal with the largest number of articles published and the journal with the largest number of citations. **(A)** Journal with the largest number of articles published. **(B)** Journals with the largest number of citations.

**Figure 4:**
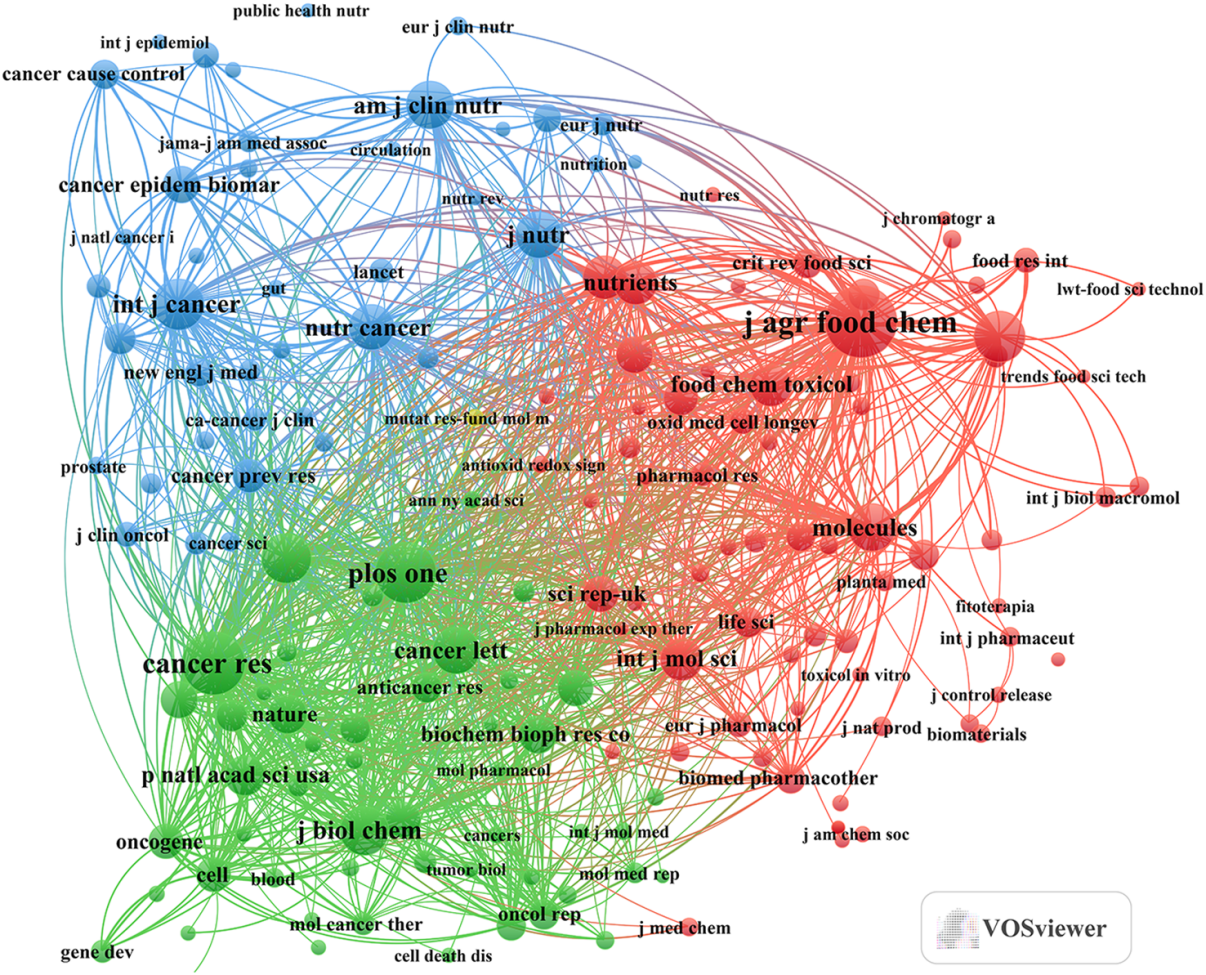
Tea in cancer co-citation journals.

The analysis of journals with the highest document counts and those garnering the most citations in this field was conducted using the R software (version 3.6.3) with the bibliometrix and ggplot2 packages. Additionally, VOSviewer (version 1.6.18) was used for co-cited journal analysis. Consequently, 4,802 papers were published across 1,296 academic journals. As illustrated in **Figure 3A** and **Table 3**, the journal with the highest number of publications was ‘Molecules’ (n=158, IF=4.6), followed by ‘Nutrients’ (n=117, IF=5.9), ‘International Journal Of Molecular Sciences’ (n=105, IF=5.6), ‘Nutrition And Cancer-An International Journal’ (n=67, IF=2.9), and ‘Food & Function’ (n=62, IF=6.1). The journals with the least published literature were ‘Nutrition And Cancer-An International Journal’ (n=67, IF=2.9) and ‘Food & Function’ (n=62, IF=6.1).Furthermore, **Figure 3B** and **Table 4** reveal that the most cited journals included ‘Journal Of Agricultural And Food Chemistry’ (n=6,265, IF=6.1), ‘Cancer Research’ (n=5,049, IF=11.2), ‘Plos One’ (n=4,824, IF=3.7), ‘International Journal Of Cancer’ (n=3,067, IF=6.4), and ‘Food Chemistry’ (n=3,486, IF=8.8). The co-cited journal map (**Figure 4**) highlights that ‘Journal of Agricultural and Food Chemistry,’ ‘Plos One,’ and ‘Nutrition And Cancer-An International Journal’ serve as prominent collaboration centers. These findings underscore a notable gap in research on tea in cancer within top-tier journals, suggesting the need for further in-depth research and exploration in this area.

**Table 3:** Top 10 journals with the most published articles.

Sources	Articles	IF	Cites
Molecules	158	4.6	3166
Nutrients	117	5.9	2729
International Journal Of Molecular Sciences	105	5.6	1
Nutrition And Cancer-An International Journal	67	2.9	2951
Food & Function	62	6.1	1537
Scientific Reports	59	4.6	1963
Antioxidants	58	7	9
Journal Of Agricultural And Food Chemistry	56	6.1	2
Frontiers In Pharmacology	51	5.6	725
Plos One	50	3.7	4824

**Table 4:** Top 10 journals with the most cited journals.

Sources	Cites	IF	Documents
Journal Of Agricultural And Food Chemistry	6265	6.1	56
Cancer Research	5049	11.2	2
Plos One	4824	3.7	50
International Journal Of Cancer	3607	6.4	32
Food Chemistry	3486	8.8	33
Carcinogenesis	3475	4.7	12
Journal of Biological Chemistry	3283	4.8	9
Molecules	3166	4.6	158
American Journal of Clinical Nutrition	3126	7.1	18
Nutrition Journal	3000	5.4	6

### 3.3 Most cited references and reference burst

The R software, utilizing the bibliometrix package, was employed to determine the 20 references with the highest citation counts within the domain of tea and its association with cancer (**Table 5**). Our findings revealed over 300 citations from 17 different journals, suggesting that a significant breakthrough in this area has yet to be achieved. The top three cited papers were: ‘Epidemiology of Esophageal Cancer,’ ‘The Immunomodulatory and Anti-Inflammatory Role of Polyphenols,’ and ‘Gallic Acid: A Versatile Antioxidant with Promising Therapeutic and Industrial Applications.’ Through a comprehensive analysis, these 20 papers can be grouped into the following themes: 1) Diet and health; 2) Plant compounds and their anticancer effects; 3) Apoptosis pathways and cancer therapy; 4) Plant extracts and their anticancer effects.

**Table 5:** Top 20 cited references related to tea in cancer.

Paper	DOI	Total Citations	TC per Year
ZHANG YW, 2013, WORLD J GASTROENTERO	10.3748/wjg.v19.i34.5598	731	66.45
YAHFOUFI N, 2018, NUTRIENTS	10.3390/nu10111618	678	113
BADHANI B, 2015, RSC ADV	10.1039/c5ra01911g	568	63.11
KIM HS, 2014, REDOX BIOL	10.1016/j.redox.2013.12.022	520	52
LI AN, 2014, NUTRIENTS	10.3390/nu6126020	491	49.1
MOHAMMAD RM, 2015, SEMIN CANCER BIOL	10.1016/j.semcancer.2015.03.001	447	49.67
IQBAL J, 2015, JAMA-J AM MED ASSOC	10.1001/jama.2014.17322	417	46.33
KOPUSTINSKIENE DM, 2020, NUTRIENTS	10.3390/nu12020457	404	101
POOLE R, 2017, BMJ-BRIT MED J	10.1136/bmj.j5024	404	57.71
DURAZZO A, 2019, PHYTOTHER RES	10.1002/ptr.6419	375	75
SATIJA A, 2017, J AM COLL CARDIOL	10.1016/j.jacc.2017.05.047	371	53
ABOTALEB M, 2019, CANCERS	10.3390/cancers11010028	366	73.2
ROY P, 2015, MATER TODAY	10.1016/j.mattod.2015.04.005	351	39
CHOUDHARI AS, 2020, FRONT PHARMACOL	10.3389/fphar.2019.01614	345	86.25
EFFERTH T, 2017, SEMIN CANCER BIOL	10.1016/j.semcancer.2017.02.009	323	46.14
MITCHELL DC, 2014, FOOD CHEM TOXICOL	10.1016/j.fct.2013.10.042	317	31.7
ESFANJANI AF, 2016, COLLOID SURFACE B	10.1016/j.colsurfb.2016.06.053	317	39.63
JAYABALAN R, 2014, COMPR REV FOOD SCI F	10.1111/1541-4337.12073	311	31.1
CHIKARA S, 2018, CANCER LETT	10.1016/j.canlet.2017.11.002	306	51
ZHENG DW, 2017, NANO LETT	10.1021/acs.nanolett.6b04060	302	43.14

**Figure 5:**
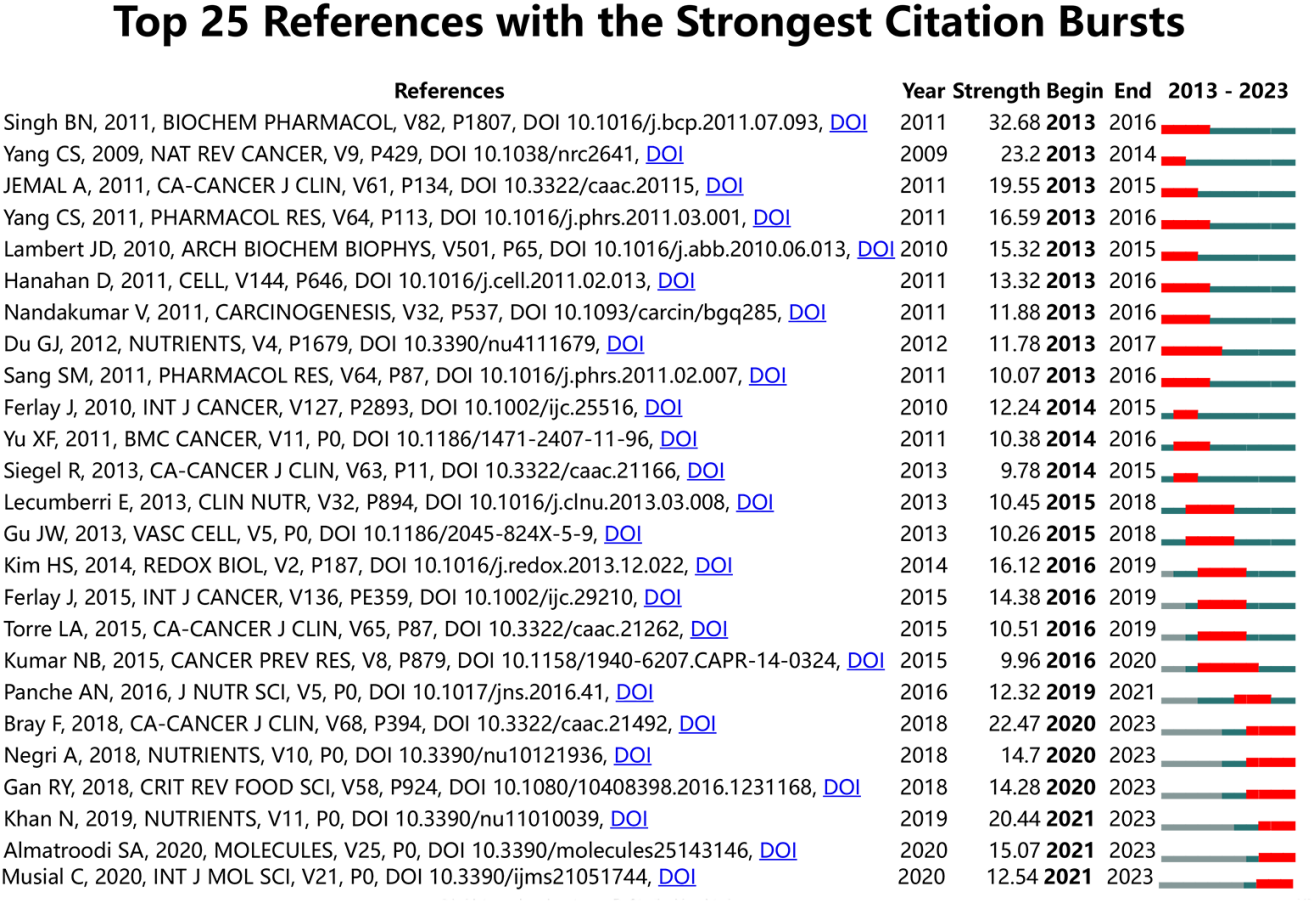
Top 25 references with the strongest citation bursts on tea in cancer.

To determine the most significant citation frequency of tea in the field of cancer, we employed Citespace in this study. **Figure 5** displays the top 25 citations with the strongest citation bursts. The citation burst intensities of these 25 documents range from 9.78 to 32.68. Among them, the top three most active documents in terms of citation bursts are: ‘Green Tea Catechin, Epigallocatechin-3-Gallate (EGCG): Mechanisms, Perspectives, and Clinical Applications (strength: 32.68),’ ‘Cancer Prevention by Tea: Animal Studies, Molecular Mechanisms, and Human Relevance (strength: 23.2),’ and ‘Global Cancer Statistics 2018: GLOBOCAN Estimates of Incidence and Mortality Worldwide for 36 Cancers in 185 Countries (strength: 22.47).’ These 25 papers can be classified according to their contents into the following categories: 1) Pharmacological effects of green tea polyphenols and cancer treatment; 2) Biological effects of polyphenols; 3) Cancer epidemiology and statistics.

When combining the top 20 most-cited papers and the top 25 papers with the strongest citation bursts in the field of tea in cancer, it becomes evident that the pharmacological effects of plant-extracted green tea polyphenols and their application in cancer therapy, as well as the bioactivity and pharmacological mechanisms of EGCG, have emerged as research hotspots in recent years. However, it is noteworthy that while green tea polyphenols and EGCG exhibit anticancer potential, their bioavailability remains relatively low. The exact pharmacological mechanism of EGCG is still under investigation. Therefore, it is reasonable to anticipate that future research in this field will prioritize the development of more efficient delivery systems, such as nanoparticles and liposomes. These systems aim to optimize the stability and availability of EGCG and green tea polyphenols *in vivo*, accompanied by a thorough examination of their pharmacological mechanisms of action.

### 3.4 Keyword clusters and evolution

Cluster analysis of keywords offers valuable insights into the focal points and trajectories of research within a specific field. In this study, a sum of 9372 keywords was obtained through the use of VOSviewer. **Table 6** presents the top 20 keywords, each with more than 200 occurrences. The most frequently appearing keyword was “Green tea” (n=1016), followed by “Cancer” (n=732), “*In-vitro*” (n=566), “Oxidative stress” (n=447), “Apoptosis” (n=434), “Expression” (n=410), “Nf-kappa-b” (n=376), “Inhibition” (n=345), and “Polyphenols” (n=324).

**Table 6:** Top 20 keywords related to tea in cancer.

Rank	Words	Count
1	Green tea	1016
2	Cancer	732
3	In-vitro	566
4	Oxidative stress	447
5	Apoptosis	434
6	Expression	410
7	Nf-kappa-b	376
8	Inhibition	345
9	Polyphenols	324
10	Growth	301
11	Tea	280
12	Mechanisms	263
13	Cells	257
14	Risk	254
15	Breast-cancer	251
16	Consumption	250
17	Black tea	230
18	Green tea polyphenol	226
19	Prostate-cancer	226
20	Green tea polyphenols	222

**Figure 6:**
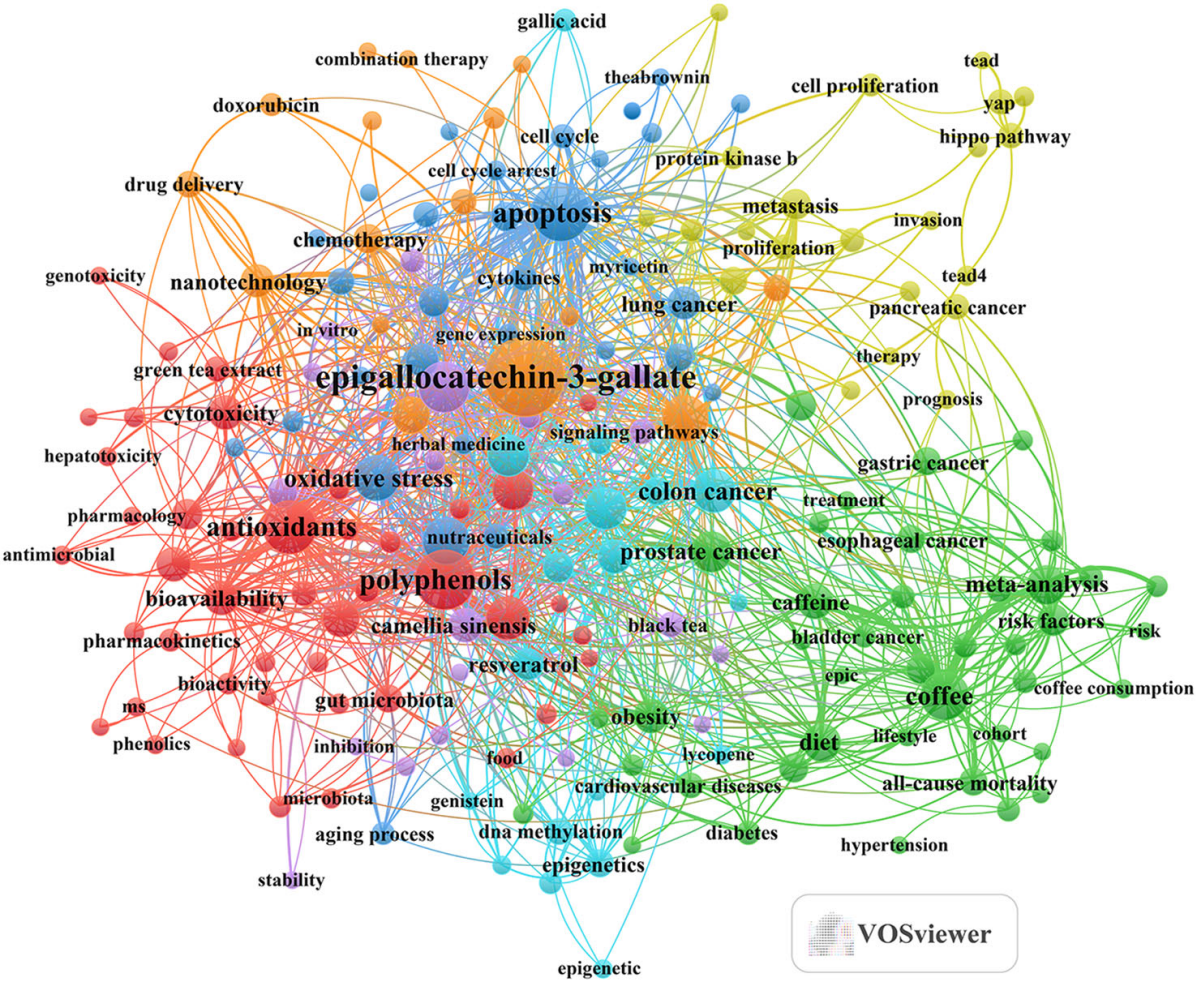
Keyword co-occurrence map of publications on tea in cancer research.

Subsequently, we selected 193 keywords based on a minimum occurrence threshold of ≥ 12, and we employed VOSviewer to create keyword clusters, as presented in **Figure 6**. Our analysis yielded seven distinct clusters. Among these clusters, the “Bioactive Compound Effects” cluster (highlighted by red dots) comprised 45 keywords, including terms such as catechins, flavonoids, polyphenols, antimicrobial properties, antioxidants, genotoxicity, cytotoxicity, and biotransformation. The “Diet and Disease” and “Research Methods” cluster (indicated by green dots) featured 41 keywords, encompassing topics like tea consumption, bladder cancer, endometrial cancer, oral cancer, gastric cancer, prostate cancer, esophageal cancer, case-control studies, dose-response relationships, and meta-analyses. In the “Cell Regulation, Cancer, and Protection” cluster (represented by blue dots), we identified 31 keywords, including apoptosis, autophagy, reactive oxygen species, the tumor microenvironment, lung cancer, and oxidative stress. The “Tumor Development and Treatment and Its Molecular Mechanisms” cluster (denoted by yellow dots) contained 22 keywords, highlighting concepts such as metastasis, invasion, yap, protein kinase b, and tead4.The “Tea and Anti-Cancer Research” cluster (marked with purple dots) consisted of 20 keywords, addressing topics like herbal tea, camellia sinensis, phenolic compounds, chlorogenic acid, clinical trials, and dietary polyphenols. The “Natural Compounds and Cancer Prevention and Their Molecular Mechanisms” cluster (illustrated by cyan dots) comprised 18 keywords, including gallic acid, resveratrol, curcumin, DNA methylation, and methylation. Lastly, the “Cancer Treatment and Drug Strategy” cluster (designated with orange dots) featured 16 keywords, encompassing terms such as doxorubicin, chemotherapy, multidrug resistance, epigallocatechin-3-gallate, catechin, and synergistic effects.

**Figure 7:**
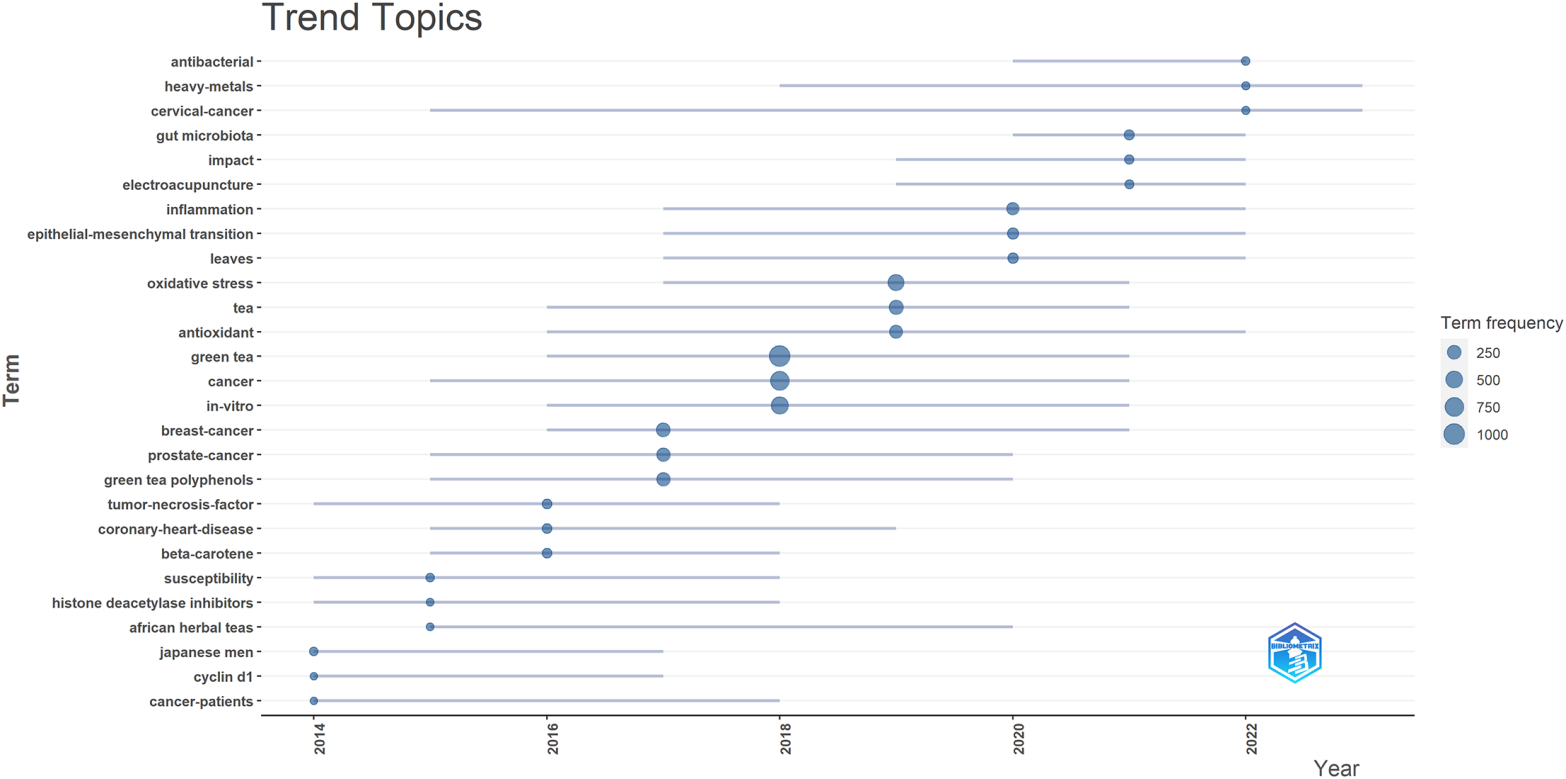
Trend topics on tea in cancer research.

Additionally, we employed the R software’s bibliometrix package to produce a trend topic map, as depicted in **Figure 7**. The Trend Theme Map proves to be a useful instrument for discerning the temporal development of particular research topics in a specific domain and analyzing the evolution of the field across different periods. Upon analyzing the trend theme map presented in **Figure 7**, we identified the current research priorities and trajectory of tea-related studies in cancer research. Notably, the prevailing hotspots in this field include cervical cancer, antioxidants, and antibacterial properties.

In summary, through keyword clustering and trend analysis, we believe that the research hotspots in tea and cancer research primarily revolve around two key aspects: 1. Investigating the antibacterial and anticancer effects of tea and tea extracts, including tea polyphenols like EGCG, and their regulatory influence on gut microbiota. 2. Exploring the molecular mechanisms underlying the use of tea extracts in cancer treatment.

## 4 Discussion

### 4.1 General information

To gain a comprehensive understanding of the focus and trajectory of tea research in the context of cancer, we undertook bibliometric and visual analyses. We examined 4,789 relevant pieces of literature spanning the period from 2013 to 2023. The study results revealed that despite fluctuations in the development of tea research within the field of cancer, an overall trend of steady growth persists. This indicates that tea’s role in cancer research continues to capture researchers’ attention and holds potential for future breakthroughs.

In the realm of tea and cancer research, China stands out as the country with the highest number of published papers, boasting 1,605 publications. This phenomenon can be attributed to China’s status as the birthplace of tea and its well-established tea culture, which provides a rich historical and cultural foundation for research in this area. It is noteworthy that among the top 15 affiliations publishing papers, only four are from China, while the rest represent affiliations from across the globe. The United States follows closely behind, with 637 papers published, securing the second position. Notably, Harvard University (184 papers) and the Egyptian Knowledge Bank (158 papers) emerge as the leading affiliations with the most significant publication records. This highlights the substantial involvement of China in tea and cancer research and the increasing global interest in this field.

A total of 4,802 papers found their place in 1,296 academic journals. Prominent among these journals are ‘Molecules,’ ‘Nutrients,’ ‘International Journal Of Molecular Sciences,’ ‘Nutrition And Cancer-An International Journal,’ and ‘Food & Function.’ It’s worth mentioning that ‘Molecules’ and ‘Plos One’ stand out as both the most-published and most-cited journals, underscoring their significance as essential publications within the realm of tea and cancer research.

### 4.2 Hotspots and development trends

By examining citation frequency, literature outbreaks, keyword clustering, and the evolution of keywords, we have clarified the focal areas and cutting-edge aspects in the realm of tea and its association with cancer. These encompass a wide range of aspects, including pharmacological effects, cancer therapy, antioxidant, and molecular mechanisms.

Firstly, the pharmacological effects and anticancer properties of tea and tea extracts constitute crucial research focal points. As evidenced by keyword clustering analysis, a substantial body of research has centered on the relationship between tea and cancer. This encompasses studies on tea’s influence on reproductive cancers (such as endometrial cancer), prostate cancer, and esophageal cancer. Tea is renowned for its health benefits attributed to the green tea catechin (GTC) molecule (27). Notably, one study has demonstrated that the catechin (GTC) molecule in green tea can impact molecular pathways, potentially halting the progression of prostate cancer (PCa). This may show specific potential for males undergoing active surveillance with low-risk PCa. Moreover, it has been established that the gut microbiota can metabolize GTCs, enhancing their absorption and efficacy within the body (28). Furthermore, research has revealed the potential of rhizopiridin extracted from sweet tea for the treatment of esophageal cancer cells. Root bark glycosides may exert their effects on cancer cells by interfering with multiple signaling pathways, including the JAK/STAT signaling pathway, MAPK signaling pathway, and apoptosis pathway. This study demonstrated that root bark glycosides antagonize the JAK2/STAT3 signaling pathway, which can inhibit the development of esophageal cancer. It has also been demonstrated that root bark glycosides promote apoptosis and inhibit autophagy in esophageal cancer cells (29).

These studies have illuminated the pharmacological effects and anticancer properties of certain teas and tea extracts, emphasizing the possible advantages of tea in the realm of cancer therapy. However, the limited bioavailability of natural plant-based products hinders their effectiveness against tumors. For instance, the chemical instability and poor bioavailability of tea polyphenols present a significant challenge for their study and application (30). While some research has initiated the exploration of delivering active components of tea (e.g., tea polyphenols) through nanocarriers, it is evident that we still have a considerable journey ahead in this field (31–33). Therefore, one of the future problems that need to be solved is the development of new delivery systems using nanocarriers to improve the bioavailability of tea and tea extracts, thus enhancing their tumor-suppressing ability (34).

Secondly, as one of the primary biological components of green tea polyphenols, EGCG has garnered significant attention due to its pharmacological mechanisms and its applications in cancer research. **Table 6** illustrates that studies in the field of tea and cancer predominantly favor green tea. This preference may be attributed to green tea’s exceptionally high content of catechins, particularly EGCG (epigallocatechin-3-gallate) and GTC (green tea catechins), in comparison to other tea varieties (18). In the context of using green tea for treating reproductive cancers, EGCG has demonstrated anti-proliferative, anti-angiogenic, and anti-metastatic properties. It also promotes apoptosis and autophagy in reproductive cancers, such as ovarian cancer cells. Furthermore, the ingestion of green tea has been linked to a reduced risk of disease progression in reproductive cancers like ovarian cancer (35). EGCG, being the primary bioactive compound in green tea, has exhibited positive effects in addressing reproductive malignancies, encompassing ovarian, cervical, and endometrial cancers (36). Research has revealed that EGCG may interact with multiple receptors and intracellular signaling pathways associated with cancer initiation and survival. These discoveries suggest that EGCG holds significant promise in cancer treatment and may function as a therapeutic drug (37, 38).

However, there remains uncertainty surrounding the outcomes of clinical trials, warranting further research to establish definitive conclusions and stronger recommendations. This is particularly pertinent in the context of green tea’s potential utilization in cancer treatment, either as a standalone therapy or in conjunction with chemotherapeutic agents (18). Furthermore, by combining the top 20 most cited papers and the top 25 papers with the most substantial citation surges in the field of tea-related cancer research, it becomes evident that the bioactivity and pharmacological mechanisms of EGCG have emerged as the focal point of recent research in this area. This underscores the importance of dedicating future efforts to elucidating these pharmacological and molecular mechanisms, thereby providing a more robust scientific foundation for the application of tea in cancer research.

Thirdly, the antioxidant and antibacterial properties of tea are also of significant interest. Plant-derived compounds, particularly antioxidants, play a vital role in neutralizing free radicals present in various disease conditions. The sustained production of free radicals within the body can lead to inflammation and potentially more severe diseases, including cancer (39). Numerous studies have demonstrated the anti-inflammatory, anti-diabetic, and anti-cancer potential of antioxidant compounds. Tea polyphenols have proven effective as antioxidants, anti-inflammatories, anti-cancer agents, and regulators of lipid metabolism. They have found widespread use in disease management (40). Furthermore, the health benefits of tea in preventing and managing various chronic diseases, such as cancer, diabetes, obesity, and cardiovascular disease, may also be attributed to tea’s antioxidant capacity (41).

Finally, within the domain of cancer-related studies, understanding the precise molecular mechanisms of tea in cancer treatment is also one of the hot topics of research. Throughout the ongoing battle against cancer, extensive efforts have led to the development of numerous anti-cancer drugs, driven by an enhanced comprehension of the molecular intricacies of this disease. However, the utilization of medications produced through chemical synthesis has not markedly elevated the overall survival rates among cancer patients. This suggests an imperative need for innovative approaches to combat cancer. Natural compounds, such as those derived from tea, show significant potential as a valuable source for the creation of innovative drugs (42). Hence, it is crucial to explore the complex molecular mechanisms through which tea can be utilized in cancer treatment, addressing the immediate research need. In recent years, several researchers have undertaken studies in this domain. Theobrowine (TB) has been found to be a major pigment and bioactive component of tea with anticancer and anti-tumor activity. Recent studies have unveiled TB’s anti-proliferative, pro-apoptotic, and tumor-suppressive impacts on HCC cells by employing a multi-targeting mechanism that engages the p53 and JNK signaling pathways (43). Furthermore, over the past few years, research has disclosed TB’s pro-senescent impact on HCC cells and its multi-targeting mechanism encompassing p53 and JNK, providing new perspectives into the inherent mechanisms driving TB’s effectiveness against HCC (44). While numerous studies have explored molecular mechanisms, the specific mechanisms underlying the significant health benefits of components such as green tea catechins remain unclear (45). This indicates the necessity for more comprehensive exploration into the molecular intricacies of tea in the context of cancer treatment.

### 4.3 Strengths and limitations

This study enhances researchers’ understanding of this field and paves the way for exploring new avenues. Nevertheless, it is crucial to recognize the constraints of this study. Firstly, our reliance solely on the WoSCC database as the data source may have resulted in overlooking certain publications. Nonetheless, the WoS database is widely considered the most suitable database for bibliometric analyses and is esteemed by researchers as a high-quality digital literature resource. Secondly, our analysis was confined to English-language publications, which may result in a relatively limited selection of sources. While acknowledging the undeniable presence of these limitations, our study furnishes a comprehensive overview of the field, underscores key research areas and emerging trends, and proves beneficial for researchers seeking a quick and comprehensive understanding of the subject. It serves as a valuable resource for researchers seeking fresh perspectives within this domain.

## 5 Conclusion

Our study reveals several key research frontiers and prominent areas of interest in the context of tea’s impact on cancer, which we have identified as follows:

- Research on the role of tea in cancer has attracted substantial international attention, with notable engagement from nations including China, the United States, India, Japan, and Italy. Extensive collaboration among these countries is evident.
- The top two journals with the highest number of publications are “Molecules” and “Nutrients.” The top two journals with the most citations are “Journal of Agricultural and Food Chemistry” and “Cancer Research.” Notably, ‘Journal of Agricultural and Food Chemistry’ is also among the top ten journals in terms of citations, signifying its representative status in the field of tea in cancer research.
- The pharmacological effects and anticancer properties of tea represent significant hotspots and trends in tea-related cancer research.
- Research focusing on the pharmacological mechanisms of natural compounds found in tea, such as polyphenols and EGCG, is another notable hotspot and research trend.
- The antioxidant and antimicrobial properties of tea are equally noteworthy research hotspots.
- Understanding the precise molecular mechanisms of tea in cancer treatment is also one of the research hotspots.

In summary, this study provides valuable perspectives on the prevailing research trends and focal points within the realm of tea research in the context of cancer. Through our analysis of current and future trends in this field, we have equipped researchers with the essential knowledge needed to gain a clear and comprehensive understanding of this domain, facilitating more efficient engagement with the field and guiding their future research directions.

## Data availability statement

The original contributions presented in the study are included in the article/supplementary material. Further inquiries can be directed to the corresponding authors.

## Author contributions

YCL: Writing – original draft. XTL: Data curation, Writing – original draft. YL: Software, Writing – original draft. ZL: Methodology, Writing – original draft. XXL: Visualization, Writing – original draft. JH: Methodology, Writing – original draft. BZ: Conceptualization, Data curation, Formal analysis, Writing – original draft. ZF: Writing – original draft, Writing – review & editing.
